# Theoretical Explanation of Upper Limb Functional Exercise and Its Maintenance in Postoperative Patients With Breast Cancer

**DOI:** 10.3389/fpsyg.2021.794777

**Published:** 2022-01-05

**Authors:** Chi Zhang, Ningning Lu, Shimeng Qin, Wei Wu, Fang Cheng, Hua You

**Affiliations:** ^1^School of Nursing, Nanjing Medical University, Nanjing, China; ^2^Department of Breast Surgery, Jiangsu Cancer Hospital, Jiangsu Institute of Cancer Research, The Affiliated Cancer Hospital of Nanjing Medical University, Nanjing, China; ^3^School of Public Health, Nanjing Medical University, Nanjing, China

**Keywords:** breast cancer, upper limb functional exercise, health action process approach, theory of planned behavior, health behavior, behavior maintenance

## Abstract

**Background:** Upper limb functional exercise (ULFE) has a positive effect on promoting the rehabilitation of upper limb function. However, little is known, about what drives postoperative patients to engage in and even maintain the advised exercises. This study integrated the health action process approach (HAPA) and the theory of planned behavior theory (TPB) to investigate the psychosocial determinants on the initiation and maintenance of ULFE in breast cancer patients. In addition, this study also tests key hypotheses relating to reasoned and implicit pathways to ULFE and its maintenance among postoperative patients with breast cancer.

**Methods:** Purposive sampling was used to recruit patients from two breast cancer wards in a provincial hospital in Jiangsu, China. Patients (*N* = 430) completed self-reported questionnaire about constructs from integrated theories concerning ULFE at an initial time point (T1): task self-efficacy, positive outcome expectations, negative outcome expectations, risk perception, attitude behavior, subjective norm, perceived behavioral control, behavioral intention, and ULFE-in hospital (ULFE-IH). Three months later (T2), patients self-reported: maintenance self-efficacy, action planning, coping planning, recovery self-efficacy, and ULFE-maintenance (ULFE-M).

**Results:** The model has a good fit (GoF = 0.48). For behavioral intention of ULFE, subjective norm (β = 0.35) and perceived behavioral control (β = 0.61) were positively directly related to behavioral intention. Regarding the initiation of ULFE, perceived behavioral control (β = 0.47) and behavioral intention (β = 0.42) had a direct positive relation to ULFE-IH. In the maintenance of ULFE, action planning (β = 0.30), coping planning (β = 0.21), maintenance self-efficacy (β = 0.32), and recovery self-efficacy (β = 0.09) all had significant positive relation on ULFE-M. In addition, maintenance self-efficacy had a significant positive association on action planning (β = 0.80), coping planning (β = 0.74), and recovery self-efficacy (β = 0.67). Coping planning was significantly predicted by behavioral intention (β = 0.07). Additionally, behavioral intention is a mediator of subjective norm (β = 0.14) and perceived behavioral control (β = 0.25) to ULFE-IH. Action planning, coping planning, and recovery self-efficacy are mediators of maintaining self-efficacy to ULFE-M (β = 0.46).

**Conclusions:** This study presents the first attempt to integrate the health behavior model in ULFE in postoperative patients with breast cancer. The study has shown that the HAPA-TPB integrated model has good applicability and effectiveness to explain and predict ULFE initiation and maintenance. Future work can be considered to develop appropriate intervention strategies based on this integrated behavioral theory.

## Introduction

In 2020, the latest data show that there are more than 2.26 million new breast cancer cases making breast cancer the most common cancer globally. Among new-onset cancers in Chinese women, there were 416,371 new cases of breast cancer, which accounted for 19.9% (World Health Organization [WHO], 2020); thus, breast cancer has become the cancer with the fastest rate of increase. Surgery, chemotherapy, and radiotherapy often produce postoperative complications and side effects, such as upper limb muscle weakness, pain, and lymphedema, which severely affect the upper limb functional activity and quality of life in patients. Studies have shown that the incidence of upper limb lymphedema is 25–40% (McLaughlin et al., 2017); the incidence of decreased muscle strength at 3 and 6 months after surgery is 23 and 19%, respectively (Zhang H. T. et al., 2020). Early postoperative upper limb functional exercise (ULFE) can ameliorate upper limb skin adhesions and edema and promote the recovery of upper limb function (Yang et al., 2018). Moreover, the risk of disease recurrence and death is reduced by 30–50% in physically active patients (Dethlefsen et al., 2016). However, some studies have shown that breast cancer patients are unable to maintain a prolonged exercise program or fail to achieve the required exercise intensity and frequency (De Groef et al., 2018). Therefore, it is critical to promote the initiation and maintenance of functional upper extremity exercise behaviors in breast cancer patients. Behavioral medicine has come to recognize that health behaviors are determined by multiple levels of influence, including personal (e.g., biological, psychological), environmental (e.g., social, physical), and policy levels (Short and Mollborn, 2015). The ULFE behavior of breast cancer patients after surgery belongs to the category of health-related behaviors. It is mainly affected by the individual (e.g., psychological perception, disease cognition, etc.) and environmental factors (e.g., hospital, important others or groups, etc.). To better explore the initiation and maintenance mechanism of ULFE behavior in breast cancer patients after surgery, this study focused on the psychosocial determinants of patients’ ULFE behavior.

Factors influencing low adherence to ULFE in breast cancer patients have been explored, and they include lack of confidence in the benefits of exercise (Hirschey et al., 2017), lack of exercise prior to diagnosis (Schmidt et al., 2017), and lack of specific knowledge about exercise (Kim et al., 2020). However, this does not provide a systematic and complete identification of the factors that influence the initiation and maintenance of behaviors. At present, behavioral theories have been widely applied within the field of ULFE behavior in breast cancer patients (Pudkasam et al., 2018). Wang et al. (2019) found that the information-motivation-behavioral skills model could predict adherence with ULFE behavior in breast cancer patients. A systematic evaluation by Rossi et al. (2018) showed that transtheoretical model and stage of change and social cognitive theory were effective interventions in patients’ ULFE behavior. The theory of planned behavior (TPB) was proposed by Ajzen (1985) based on the theory of reasoned action (Ajzen, 1985). The theory posits that behavioral intention is the most direct factor affecting behavior occurrence, and behavioral intention is determined by attitude behavior (including behavioral beliefs and evaluation of behavioral outcomes), subjective norm (including normative beliefs and motivation to comply), and perceived behavioral control (including control beliefs and perceived power) (Ajzen, 1985). In addition, behavior can also be determined by perceived behavioral control. TPB has a good guiding role in the interpretation, prediction, and intervention of behaviors. Studies have shown that TPB can explain the cesarean section behavior of Chinese pregnant women (Sun et al., 2020), and predict binge drinking behavior (Lawental et al., 2018), and develop intervention strategies to reduce alcohol consumption (Norman et al., 2018), etc. However, it still have some limitations in using these theories to explain the key factors of behavioral variables (Pudkasam et al., 2018), and nearly 50% of behavioral intention and variance of behaviors have not been explained (Shen et al., 2010). In some cases, the existing theories, such as TPB, need to be integrated or compensated into the new theoretical model with the retained original core components.

Most of the studies in this category focus on the motivational phase of exercise behavior and few studies have assessed relevant influences during the volitional phase of the behavior (Kindred et al., 2020). It is worth noting that functional exercise is a behavior that requires persistence and that it is important to identify factors influencing the maintenance of functional exercise behavior. Thus, it is necessary to develop relevant behavioral theories to guide ULFE behaviors and their maintenance. The health action process approach (HAPA) is a new stage theory of health behavior proposed by Schwarzer (1992). It has been used in numerous health behavior researches to identify psychological determinants of health behaviors (Duan et al., 2018; Liang et al., 2019; Zhang et al., 2019a). The theory includes the motivational phase and volitional phase. In the motivational phase, an individual’s intention to implement a certain behavior is influenced by task self-efficacy (It is an optimistic belief), outcome expectations (It refers to whether a person believes a certain behavior will cause a desired change), and risk perception (It refers to the perceived health threat or concern that requires action mobilization) (Schwarzer, 2016). The volitional phase consists of action planning to specify when, where, and how to perform the behavior, coping planning how to circumvent anticipated barriers (Schwarzer, 2016). The HAPA model suggests that behavior, as well as depending on the action and coping planning, also depends on perceived ability in adhering to the behavior (maintenance self-efficacy) and in coping after ending the behavior (recovery self-efficacy) (Schwarzer, 2008).

HAPA is a two-level model, which integrates relevant variables in continuity and stage theory, and can systematically explain the whole process of a health behavior from behavioral motivation to intention, plan, behavior initiation, and maintenance (Zhang et al., 2019b). It has been suggested that HAPA may be a relevant model for studying physical exercise in breast cancer patients. However, more studies are required for its validation (Paxton, 2016). HAPA and TPB are structurally similar in that they both include intention and behavior in their core concepts, but differ in the variables that precede behavioral intention (i.e., task self-efficacy, outcome expectations, and risk perception for HAPA precede behavioral intention; attitude behavior, subjective norm, and perceived behavioral control for TPB). The difference in this process is that HAPA has mediating variables from behavioral intention to behavior (including action planning and coping planning) and includes the role of both maintenance self-efficacy and recovery self-efficacy, whereas the intention-to-behavioral gap has not been addressed by TPB. From this, it can be seen that HAPA may well compensate for TPB’s lack of mediating variables. TPB provides more supplements for the antecedents of intention formation; therefore, combining both models could give a more comprehensive explanation of the motivational phase. Some scholars have conducted preliminary studies on the integrated HAPA-TPB model. Shen et al. (2010) first proposed the integrated HAPA-TPB model in 2010 by combining the elements of HAPA and TPB and the characteristics of adult exercise behavior. Ever since HAPA-TPB was proposed in the field of exercise behavior, scholars have also integrated HAPA and TPB to conduct studies in other behaviors. Cheng and Zhong (2016) used HAPA as the main model and integrated HAPA’s task self-efficacy, outcome expectations, risk perception, maintenance self-efficacy, recovery self-efficacy, and coping planning and TPB’s attitude behavior and subjective norm to apply them to miner’s unsafe behaviors. Zhang C. Q. et al. (2020) used the HAPA structure as the main model and integrated HAPA’s task self-efficacy, maintenance self-efficacy, action planning, and coping planning and TPB’s attitude behavior, subjective norm, and perceived behavioral control to apply them to hand-washing and sleep hygiene in college students, achieving the better effect. Therefore, the HAPA-TPB integrated model has a good application in some health behaviors of the Chinese population. It provides reference evidence for its application in the health behavior management of breast cancer patients and fills the research gap in Chinese breast cancer patients. In this study, all constructs of HAPA and TPB were integrated to form the HAPA-TPB model. In order to explores the factors that influence the initiation and maintenance of functional upper extremity exercise behaviors in breast cancer patients based on the integrated model and to provide a theoretical basis for the development of multidimensional and individualized interventions.

## Aims of the Current Study

The main research aim was to explore the relevant factors of initiation and maintenance of ULFE behavior in breast cancer patients. The main hypothesis is as follows: In the motivational phase, task self-efficacy, positive outcome expectations, negative outcome expectations, risk perception, attitude behavior, subjective norm, perceived behavioral control with behavioral intention are positively correlated. Task self-efficacy is positively related to maintenance self-efficacy. Perceived behavioral control and behavioral intention are positively related to upper limb functional exercise-in hospital (ULFE-IH). Perceived behavioral control is positively related to upper limb functional exercise-maintenance (ULFE-M) (home-based ULFE). In the volitional phase, behavioral intention is positively correlated with action planning and coping planning. Maintenance self-efficacy is positively correlated with action planning, coping planning, recovery self-efficacy, and ULFE-M. Action planning, coping planning, and recovery self-efficacy are positively correlated with ULFE-M. The second purpose is to explore the mediating role of behavioral intention in the HAPA-TPB integrated model on the initiation of ULFE behavior and the mediating effects of recovery self-efficacy, action planning, and coping planning on the maintenance of ULFE behavior.

## Methodology

### Study Design and Participants

The purposive sampling method was used to conduct the longitudinal study in two breast surgery wards of a cancer hospital in Jiangsu, China. Enrollment and follow-up were conducted from August 2020 to September 2021. Inclusion criteria were as follows: those who were female patients with primary breast cancer who had undergone surgery; had first and unilateral onset; had clear consciousness; and provided consent to participate and cooperate with the study. Exclusion criteria were as follows: those who were unaware of their condition; were ≤ 18 years; had preoperative upper limb dysfunction; had comorbid severe diseases; had serious postoperative complications; or had distant metastases.

The questionnaire survey was divided into two times (T1: about 3 days after surgery, T2: about 3 months after surgery). The first survey was conducted in the form of a face-to-face. The survey content was as follows: task self-efficacy, positive outcome expectations, negative outcome expectations, risk perception, attitude behavior, subjective norm, perceived behavioral control, behavioral intention, and ULFE-IH. Each participant took about 10 min, covering the period from August 2020 to June 2021; The second follow-up survey took the form of telephone follow-up. The survey content was as follows: maintenance self-efficacy, action planning, coping planning, recovery self-efficacy, and ULFE-M. Each subject took about 8 min, covering the period from November 2020 to September 2021.

In this study, the sample size was 5–10 times the number of variables (Ni et al., 2010). It was estimated based on the HAPA-TPB integrated model questionnaire with the most independent variables. The HAPA-TPB questionnaire involved 65 independent variables in total. Considering that 10% of the questionnaires were invalid, at least 361 cases need to be collected for this study. A total of 430 questionnaires were distributed and received in this study, with a response rate of 100%. After the first survey, 9 people to have the same answer for each item in the questionnaire. 8 individuals did not meet the inclusion and exclusion criteria; 5 were missing more than two-thirds of the information in the questionnaire. The questionnaires of the above 22 subjects were invalid, so the second survey could not be conducted. During the second survey, only 7 people refused follow-up or could not be connected, and the dropout rate was only 1.72% (<5%). Therefore, 401 valid questionnaires were collected, with a validity rate of 93.3%.

### Measures

The questionnaire consists of three parts: demographic information, independent variables, and dependent variables. Demographic information includes age, education level, financial status, employment status, and type of social medical insurance. The last two parts were designed according to the integrated model, which contains 14 dimensions. Each item of the dimensions was designed on a 5-point Likert scale, with scores of 1–5 assigned on a scale from *not at all* to *fully compliant*, and 5–1 for the reverse items, respectively. All observed variables in the measurement model were reflective indicators and factor loadings for the factors were > 0.7 (Urbach and Ahlemann, 2010). After the deletion of several items with poor reliability test results, the ULFE behavior questionnaire for breast cancer patients was finalized. The total Cronbach’s alpha coefficient of the questionnaire is 0.96. The Kaiser-Meyer-Olkin value for the questionnaire was 0.95, with a Bartlett’s test of sphericity *P* < 0.001. The questionnaire is presented in Supplementary Table 1.

Independent variables contain 12 dimensions. The Cronbach’s alpha coefficient of each dimension is greater than 0.7, ranging from 0.82 to 0.96. In the motivational phase: Task self-efficacy was assessed with 5 items such as “I can start the upper limb functional exercise, even if I want to rest after surgery.” Positive outcome expectations were measured with 4 items such as “If I do the upper limb functional exercise, it can lighten the burden on the family.” Negative outcome expectations were assessed with 2 items such as “If I do the upper limb functional exercise, it needs to take time and energy.” Risk perception was measured with 5 items such as “If you don’t do the upper limb functional exercise, what do you think is the likelihood of postoperative complications? Breast cancer-related lymphedema.” Attitude behavior was assessed with 4 items such as “I think the time and effort spent on the upper limb functional exercise is worth it.” Subjective norm was measured with 5 items such as “I am willing to do upper limb functional exercise as instructed by the medical staff.” Perceived behavioral control was assessed with 5 items such as “I have enough time and energy to do upper limb functional exercise every day.” Behavioral intention was assessed with 4 items such as “I ask my family to push me to do upper limb functional exercise.” In the volitional phase: Maintenance self-efficacy was assessed with 6 items such as “I can maintain my upper limb functional exercise, even if I have to keep trying.” Recovery self-efficacy was measured with 5 items such as “I’ve postponed a couple of times upper limb functional exercise, and I believe I can resume them.” Action planning was measured with 4 items such as “I have planned the time for upper limb functional exercise.” Coping planning was assessed with 5 items such as “I have a way to deal with situations that might prevent an upper limb exercise program.”

The Cronbach’s alpha coefficients of ULFE-IH and ULFE-M are 0.90 and 0.72, respectively. ULFE-IH was assessed with 3 items such as “I learned the specific methods and skills of upper limb functional exercise.” ULFE-M was measured with 3 items such as “I often observe the recovery of upper limb function.”

### Statistical Analysis

In order to control the common method bias (Zhou and Long, 2004), this study carried out the procedural control of the questionnaire, such as group discussion and pre-survey. At the same time, the collected data were tested by Harman’s single factor test. Unrotated exploratory factor analysis results extracted a total of 8 factors (>1) with characteristic roots more than 1, and the maximum factor variance explanation rate was 33.08% (<40%), so there was no serious common method bias in this study. Data was initially treated for missing data using the multiple imputation approach. In this study, P-P plots were used to test the normality of the variables and descriptive statistics were analyzed using frequencies and composition ratios, means and standard deviations, and medians and interquartile ranges. Since this study is an integrated model exploration and the model is complex, partial least squares structural equation modeling was used to test the relationship between the latent variables. The structural equation model contains a measurement model and a structural model. Cronbach’s α (>0.7), composite reliability (CR) (>0.7), and average variance extracted values (AVE) (>0.5) were used in the measurement model to evaluate the consistency of the data, and the Fornell–Larcker criterion was used to determine whether there was differential validity between the constructs (i.e., the root value of AVE was greater than the correlation between the constructs) (Urbach and Ahlemann, 2010). We tested the overall model fit using the goodness-of-fit (GoF) value (cut-offs of 0.1, 0.25, 0.36, respectively, for weak, medium, and strong) (Wetzels et al., 2009). The 5,000 iteration bootstrapping algorithm was used to test the significance of the relationship between the potential variables. *R*^2^ and *Q*^2^ (>0) were used to test the explanatory and predictive power of the structural model. *R*^2^-values of 0.19, 0.33, and 0.67, as suggested by Urbach and Ahlemann (2010), correspond to small, medium, and large explanatory powers, respectively. *f*^2^-values of 0.02, 0.15, and 0.35, as suggested by Cohen (1988), correspond to weak, moderate, strong effects. All data were analyzed using SPSS 25.0 and Smart PLS 3.3.1 software and test significance was a two-tailed alpha = 0.05.

## Results

### Basic Information

The characteristics of the subjects (*N* = 401) are listed in Table 1. All the subjects are females. The mean age of the subjects was 52.85 ± 10.03 years. Ninety-two percent of the subjects were married, 35.2% of them had an education level of junior high school, and 29.4% were retirees. The median average monthly household income was 5,500 (CNY). With regard to the type of social medical insurance, 200 (49.9%) had urban employee basic medical insurance and 179 (44.6%) had urban and rural resident basic medical insurance.

**TABLE 1:** Sociodemographic characteristics (*n* = 401).

Variables		n	%
Age (year) (Mean ± SD)		52.85	10.03
Marital status	Married	369	92.0
	Single	32	8.0
Education level	Primary school and below	109	27.2
	Junior high school	141	35.2
	High school	56	14.0
	Junior college	52	13.0
	Bachelor degree and above	43	10.7
Employment status	In-service staff	79	19.7
	Unemployed	102	25.4
	Retiree	118	29.4
	Farmer	58	14.5
	Others	44	11.0
Average monthly household income (CNY) [Median ± (Q3-Q1)]^a^		5500.0	5166.5
Type of social medical insurance	UEBMI	200	49.9
	URRBMI	179	44.6
	Others	19	4.7
	Uninsured	3	0.7

*^a^The 27 missing data in average monthly household income were interpolated by multiple imputation approach; UEBMI, urban employee basic medical insurance; URRBMI, urban and rural resident basic medical insurance.*

### Measurement Model

Means, standard deviations, Cronbach’s ɑ, and bivariate correlations for all model variables are presented in Table 2. Supplementary Table 2 shows the results of the measurement models for each dimension. The factor loading for each factor was > 0.7 except for one item in coping planning, which had an acceptable factor loading of 0.69 (close to 0.7). The Cronbach’s ɑ coefficients for each dimension ranged from 0.72 to 0.96, CR values ranged from 0.83 to 0.97, and AVE values ranged from 0.63 to 0.89. The above results indicate that the measurement models have good reliability and convergent validity. Supplementary Table 3 shows the discriminant validity of the measurement models, and the root values of AVE are all greater than the correlation coefficients between the constructs, indicating that there is good discriminant validity between the measurement models.

**TABLE 2:** Upper limb functional exercise: Means, standard deviations (SDs), Cronbach’s α, and bivariate correlations for variables of the HAPA-TPB integrated model.

Constructs	Means	SDs	Cronbach’s α	TSE	POE	NOE	RP	MSE	AP	CP	RSE	AB	SN	PBC	BI	ULFE-IH
Task self-efficacy (TSE)	16.94	5.28	0.92													
Positive outcome expectations (POE)	14.46	3.88	0.91	0.70												
Negative outcome expectations (NOE)	6.43	2.29	0.87	0.51	0.53											
Risk perception (RP)	16.15	6.01	0.96	0.58	0.58	0.54										
Maintenance self-efficacy (MSE)	18.85	5.74	0.93	–0.05	–0.04	–0.09	–0.07									
Action planning (AP)	12.11	4.08	0.92	–0.02	–0.05	–0.10	–0.07	0.80								
Coping planning (CP)	13.79	5.03	0.92	0.03	0.01	–0.12	–0.04	0.75	0.75							
Recovery self-efficacy (RSE)	15.62	5.29	0.93	–0.10	–0.10	–0.08	–0.09	0.67	0.62	0.57						
Attitude behavior (AB)	14.81	3.43	0.82	0.69	0.73	0.45	0.58	–0.05	–0.07	0.01	–0.07					
Subjective norm (SN)	18.80	4.45	0.89	0.66	0.70	0.52	0.58	0.05	0.05	0.08	–0.03	0.76				
Perceived behavioral control (PBC)	17.03	5.17	0.92	0.68	0.66	0.41	0.53	0.07	0.07	0.10	0.002	0.74	0.79			
Behavioral intention (BI)	14.18	4.31	0.93	0.62	0.62	0.39	0.52	0.14	0.14	0.17	0.05	0.67	0.80	0.86		
ULFE-in hospital (ULFE-IH)	10.77	3.09	0.90	0.71	0.63	0.42	0.53	0.05	0.07	0.12	0.02	0.70	0.78	0.82	0.81	
ULFE-maintenance (ULFE-M)	9.53	2.82	0.72	–0.07	–0.10	–0.20	–0.14	0.77	0.77	0.72	0.61	–0.14	–0.04	0.03	0.07	0.02

*ULFE, Upper Limb Functional Exercise.*

### Structural Model

Supplementary Table 4 shows the measurements of the structural model. The model explained 72% of the variance in ULFE behavior initiation and 68% of the variance in behavior maintenance in breast cancer patients, indicating that the HAPA-TPB has strong explanatory power and practical value for ULFE behavior in breast cancer patients. The *Q*^2^ are > 0, indicating strong predictive relevance of the model. The GoF was 0.48, showing that the fitness of the model was good.

**FIGURE 1:**
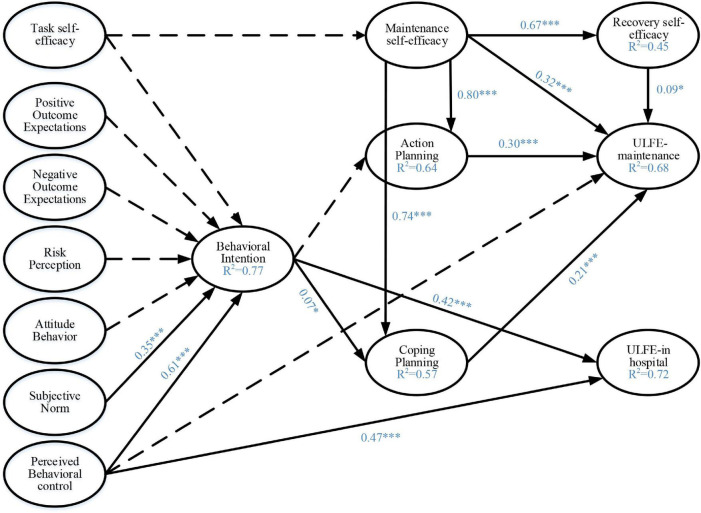
Standardized path coefficients for the structural equation model explaining and predicting upper limb functional exercise behavior based on the HAPA-TPB integrated model. *P < 0.05; ^***^P < 0.001.

The path analysis of latent variables in the integrated model and effect size *f*^2^ are shown in Table 3 and Figure 1. For behavioral intention of ULFE, subjective norm [β(95%CI) = 0.35 (0.21, 0.49), *P* < 0.001] and perceived behavioral control [β(95%CI) = 0.61 (0.50, 0.70), *P* < 0.001] were positively related to behavioral intention. For ULFE behavior initiation, perceived behavioral control [β(95%CI) = 0.47 (0.34, 0.59), *P* < 0.001] and behavioral intention [β(95%CI) = 0.42 (0.29, 0.54), *P* < 0.001] were positively correlated with ULFE-IH. For behavioral maintenance of ULFE, action planning [β(95%CI) = 0.30 (0.20, 0.40), *P* < 0.001], coping planning [β(95%CI) = 0.21 (0.11, 0.32), *P* < 0.001], maintenance self-efficacy [β(95%CI) = 0.32 (0.19, 0.43), *P* < 0.001], and recovery self-efficacy [β(95%CI) = 0.09 (0.01, 0.18), *P* < 0.05] were all positively associated with ULFE-M. Maintenance self-efficacy was positively related to action planning [β(95%CI) = 0.80 (0.76, 0.83), *P* < 0.001], coping planning [β(95%CI) = 0.74 (0.69, 0.79), *P* < 0.001], and recovery self-efficacy [β(95%CI) = 0.67 (0.60, 0.74), *P* < 0.001]. Behavioral intention [β (95%CI) = 0.07 (0.01, 0.14), *P* < 0.05] was positively related to coping planning. The remaining paths were not significant (*P* > 0.05).

**TABLE 3:** Path analysis results.

Relationships	Standardized coefficient (β)	*f* ^2^	95% CI		*P*-values
			Lower	Upper	
Task self-efficacy - > Behavioral intention	0.003	−	–0.07	0.09	0.94
Positive outcome expectations - > Behavioral intention	0.01	−	–0.06	0.08	0.74
Negative outcome expectations - > Behavioral intention	–0.04	−	–0.10	0.02	0.17
Risk perception - > Behavioral intention	0.04	−	–0.01	0.10	0.13
Attitude behavior - > Behavioral intention	–0.06	−	–0.15	0.04	0.24
Subjective norm - > Behavioral intention	0.35	0.14	0.21	0.49	***
Perceived behavioral control - > Behavioral intention	0.61	0.48	0.50	0.70	***
Task self-efficacy - > Maintenance self-efficacy	–0.05	−	–0.15	0.04	0.30
Behavioral intention - > Action planning	0.03	−	–0.03	0.09	0.28
Behavioral intention - > ULFE-in hospital	0.42	0.17	0.29	0.54	***
Behavioral intention - > Coping planning	0.07	0.01	0.01	0.14	*
Perceived behavioral control - > ULFE-maintenance	–0.04	−	–0.10	0.02	0.19
Perceived behavioral control - > ULFE-in hospital	0.47	0.21	0.34	0.59	***
Maintenance self-efficacy - > Coping planning	0.74	1.26	0.69	0.79	***
Maintenance self-efficacy - > Action planning	0.80	1.74	0.76	0.83	***
Maintenance self-efficacy - > Recovery self-efficacy	0.67	0.83	0.60	0.74	***
Recovery self-efficacy - > ULFE-maintenance	0.09	0.01	0.01	0.18	*
Maintenance self-efficacy - > ULFE-maintenance	0.32	0.09	0.19	0.43	***
Action planning - > ULFE-maintenance	0.30	0.09	0.20	0.40	***
Coping planning - > ULFE-maintenance	0.21	0.05	0.11	0.32	***

*P < 0.05; ***P < 0.001.

*ULFE, Upper Limb Functional Exercise. f^2^ represents the influence of exogenous variables on endogenous variables.*

### Mediating Effects

A mediator effects analysis revealed that subjective norm can positively correlated ULFE-IH [β(95%CI) = 0.14 (0.07, 0.26), *P* < 0.01] indirectly through behavioral intention. Perceived behavioral control can directly positively correlated ULFE-IH [β(95%CI) = 0.47 (0.33, 0.59), *P* < 0.001] and can also positively correlated ULFE-IH [β(95%CI) = 0.25 (0.19, 0.34), *P* < 0.001] through behavioral intention. Maintenance self-efficacy through action planning/coping planning/recovery self-efficacy was indirectly and positively correlated with ULFE-M [β(95%CI) = 0.46 (0.36, 0.56), *P* < 0.001]. Maintenance self-efficacy was positively correlated with ULFE-M [β(95%CI) = 0.32 (0.20, 0.43), *P* < 0.001]. Thus, behavioral intention acted as full mediators in the subjective norm - > ULFE-IH pathway. Behavioral intention played a partial mediator role in the perceived behavioral control - > ULFE-IH pathway. Action planning, coping planning, and recovery self-efficacy acted as partial mediators in all three pathways from maintenance self-efficacy to ULFE-M (Table 4).

**TABLE 4:** Mediating effect.

Path	Direct effects	Indirect effects	Total effects
	β (95%CI)	β (95%CI)	β (95%CI)
Subjective norm - > Behavioral intention - > ULFE-in hospital	−	0.14 (0.07,0.26)**	0.14 (0.07,0.26)**
Perceived behavioral control - > Behavioral intention - > ULFE-in hospital	0.47 (0.33,0.59)***	0.25 (0.19,0.34)***	0.72 (0.63,0.79)***
Maintenance self-efficacy - > Action planning/Coping planning/Recovery self-efficacy - > ULFE-maintenance	0.32 (0.20,0.43)***	0.46 (0.36,0.56)***	0.77 (0.74,0.80)***

**P < 0.01; ***P < 0.001.ULFE, Upper Limb Functional Exercise.

## Discussion

Since surgery remains the main modality for breast cancer, post-surgery rehabilitation, especially restricted upper limb function recovery, is still a critical health problem in this patient population (Zhou et al., 2019). To date, there is a dearth of research that has investigated the psychological correlation between initiation and maintenance of ULFE behavior in breast cancer patients. The aim of this study was to examine the feasibility and applicability of the integrated HAPA-TPB model on ULFE behavior in breast cancer patients. The current study confirmed that the HAPA-TPB could explain and predict the initiation and maintenance of ULFE behavior in breast cancer patients.

The model includes the initiation and maintenance stages of the ULFE behavior. In the initiation of behavior, the results of the integrated model showed that subjective norm and perceived behavioral control were indirectly related to ULFE-IH through behavioral intention. Moreover, perceived behavioral control can have a direct relation on ULFE-IH. In addition, task self-efficacy, outcome expectations, risk perception, and attitude behavior did not significantly predict intention. In terms of maintenance of behavior, the results suggest that maintenance self-efficacy, action planning, coping planning, and recovery self-efficacy is directly related to ULFE-M. Maintenance self-efficacy can also have an indirect related on ULFE-M through action planning, coping planning, and recovery self-efficacy. However, there was no moderating effect of action planning and coping planning on the relationship between intention and ULFE-M.

Current findings on the initiation of behavior are inconsistent with some previous studies and the theoretical hypothesis (Duan et al., 2018; Zhang et al., 2019b; Liddelow et al., 2020; Rhodes et al., 2021). Specifically, task self-efficacy, outcome expectations, risk perception, and attitude behavior did not significantly predict intention. There may be three reasons: First, breast cancer patients place a high value on the recovery of upper limb function. Second, breast cancer patients were able to follow the norms for ULFE as requested by their health care providers in the early postoperative period. Third, the patient was first diagnosed with breast cancer, low knowledge of the disease, and a lack of understanding of the duration and precautions for performing ULFE. The results of the integrated model showed that perceived behavioral control are determinants of behavioral intention and ULFE-IH. This is similar to the previous TPB studies on other health behaviors (Strong et al., 2018; Liddelow et al., 2020). Therefore, we propose the following recommendations to enhance perceived behavioral control: Healthcare providers should (1) provide training to patients regarding breast cancer and functional exercises for the upper extremities, and (2) provide patients with access to appropriate resources and improve their ability to handle problems. The results also found that subjective norm indirectly affected ULFE-IH through behavioral intention, which is consistent with the findings of Blanchard et al. (2002). Subjective norm refers to the social pressure perceived by an individual in deciding whether to perform a particular behavior (St-Pierre et al., 2015). It reflects the influence of important others or groups on an individual’s behavioral decisions. Therefore, we suggest that health care providers increase their efforts to popularize knowledge on breast cancer and postoperative rehabilitation to increase the social pressure on patients. The patient’s family and friends should also be mobilized to participate in ULFE behavior. Therefore, subjective norm and perceived behavioral control are factors that should be fully considered when developing interventions to promote behavioral intention and exercise behaviors for ULFE in breast cancer patients.

Regarding the maintenance of behavior of the proposed integrated model, findings of the current study are consistent with previous meta-analyses (Zhang et al., 2019b). Specially, maintenance self-efficacy and recovery self-efficacy are important determinants of the maintenance of ULFE behavior in breast cancer patients. It was noted that self-efficacy is needed throughout the behavior change process because different challenges arise when people move from one stage to the next (Lippke and Plotnikoff, 2014). Moreover, Hsu et al. (2011) also showed that the level of self-efficacy in breast cancer patients was a significant predictor of the maintenance of exercise behaviors and that the implementation of self-efficacy interventions helped to improve the maintenance of ULFE behavior in patients. Therefore, factors such as patients’ self-efficacy and stages of action should be fully considered when developing health behavior intervention strategies so that interventions can be implemented more precisely to promote the maintenance of exercise behaviors in patients.

The current study found that action planning and coping planning did not have a mediating or moderating role in the behavioral intention - > ULFE-M pathway. It is inconsistent with previous researches (Cowie et al., 2018; Lin et al., 2018). There may be two reasons for this: First, ULFE were strictly supervised by health care providers during the hospitalization of breast cancer patients after surgery. Second, during patient hospitalization, the health care provider delivers education and instruction on ULFE behavior to the patient. However, it is noteworthy that the planning factor is a mediator variable for the maintenance self-efficacy - > ULFE-M pathway. Xu et al. (2020) also found that maintenance self-efficacy indirectly predicted physical activity through planning. Another study showed that the initiation and maintenance of behaviors require action planning and coping planning (Paxton, 2016). A study has also pointed out that social support is an important factor for patients to adopt and maintain healthy behaviors after discharge (Duan et al., 2018). Therefore, future studies should consider incorporating social support into the model to verify its mediating role in behavioral intention to behavior. ULFE in breast cancer patients is a process that requires long-term maintenance, a gradual progress, and complex movements, and many patients slack off or even stop exercising in the middle and late stages of exercise. Even if the ULFE behavior is maintained every day, many patients only perform simple upper limb lifting or finger climbing wall movements. Moreover, many patients report that they do not have a regular exercise program. A qualitative study also showed that as patients prefer less restriction and more freedom, they often do not have an exercise schedule and simply perform it whenever it is convenient in their daily lives (Tsai et al., 2018). It has been suggested that the planning factor in HAPA may be better applied to behaviors that require long-term maintenance and those that require complex steps (Lao et al., 2021), and this study validates this finding. This suggests that action planning and coping planning are important mediators of the maintenance of ULFE behavior in breast cancer patients. Therefore, feasible interventions should be created to increase patients’ willingness to develop action planning, and also to improve patients’ ability to cope with difficulties in performing health behaviors in order to promote behavior maintenance.

### Limitations and Future Directions

This study has the following limitations: First, it was conducted in only one hospital, the sample size was limited, and the heterogeneity of the study population may be relatively low. Also, purposive sampling has a limitation for the sample representativeness. Multicenter large sample studies may be conducted in the future for further validation of the results. Second, in this study, there are some differences between on-site face-to-face questionnaires and telephone follow-up. In addition, all variables were collected by self-reported measures, which may cause recall bias and measurement error. It may have some effects on the study results. Third, only females were included, and demographic characteristics were not included as covariables in the model. It is suggested that the influence of demographic characteristics such as gender should be fully considered in future studies. Fourth, past behavior/habit strength and more psychosocial constructs (e.g., self-monitoring/action control, perceived social support, social-environmental cues) could be systematically examined in the future. Despite these limitations, this study confirmed the feasibility and applicability of the integrated HAPA-TPB model, thus providing a basis for the strategy’s development of a multidimensional and phased intervention for ULFE behavior after breast cancer surgery.

## Conclusion

The integrated HAPA-TPB model has good applicability and validity in ULFE behavior in breast cancer patients. Further, the model can better explain and predict the initiation and maintenance of ULFE behavior. Mainly, subjective norm and perceived behavioral control are important relation factors on the initiation of ULFE behavior. Behavioral intention is a mediating variable on the initiation of ULFE behavior. Maintenance self-efficacy is a direct relation factor on the maintenance of ULFE behavior. Action planning, coping planning, and recovery self-efficacy are both direct and indirect relation factors on the maintenance of ULFE behavior. Appropriate intervention strategies for ULFE can be formulated on the basis of this behavioral integration model in order to reduce the incidence of postoperative complications and improve the quality of life of breast cancer patients.

## Data Availability Statement

The raw data supporting the conclusions of this article will be made available by the authors, without undue reservation.

## Ethics Statement

Permission for this study was obtained from all participants. This study was approved by the Ethics Review Committee of Nanjing Medical University [(2020)591].

## Author Contributions

HY and FC designed the study and critically reviewed, and commented the manuscript. HY revised the manuscript. CZ and NL participated in data collection and analysis, performed the final statistical analyses, and prepared the first version of the manuscript. SQ and WW collected the data and literature materials. All authors contributed to the article and approved the submitted version.

## Conflict of Interest

The authors declare that the research was conducted in the absence of any commercial or financial relationships that could be construed as a potential conflict of interest.

## Publisher’s Note

All claims expressed in this article are solely those of the authors and do not necessarily represent those of their affiliated organizations, or those of the publisher, the editors and the reviewers. Any product that may be evaluated in this article, or claim that may be made by its manufacturer, is not guaranteed or endorsed by the publisher.
