# Supplementary material for: Theoretical Explanation of Upper Limb Functional Exercise and Its Maintenance in Postoperative Patients With Breast Cancer
Source: Front Psychol. 2022 Jan 5;12:794777. doi: 10.3389/fpsyg.2021.794777 (PMC8766984; doi:10.3389/fpsyg.2021.794777)
Supplement: Supplementary file 1 [file Table_1.DOCX]

Supplementary Material

# Supplementary Tables

**Supplementary Table 1.** HAPA-TPB questionnaire

| Constructs | Item labels | Item | Decision |
| --- | --- | --- | --- |
| Task self-efficacy (TSE)  (I can start the upper limb functional exercise under the following conditions:) | TSE1 | Even if I wanted to rest after surgery | Keep |
|  | TSE2 | Even if the upper limb functional exercise is a hard thing | Keep |
|  | TSE3 | Even if the specific methods and skills of upper limb functional exercise are complex | Keep |
|  | TSE4 | Even if I have to push myself to do upper limb functional exercise | Keep |
|  | TSE5 | Even if I have to force myself to do upper limb functional exercise | Keep |
| Positive Outcome Expectations (POE)  (If I do the upper limb functional exercise) | POE1 | It can lighten the burden on the family | Keep |
|  | POE2 | It will reduce my visits to the doctor | Keep |
|  | POE3 | It will improve my quality of life | Keep |
|  | POE4 | It will speed up the recovery of upper limb function on the affected side | Keep |
| Negative Outcome Expectations (NOE)  ( If I do the upper limb functional exercise) | NOE1 | It will take time and energy | Keep |
|  | NOE2 | It will make me make many great efforts | Keep |
| Risk Perception (RP)  (If you don’t do the upper limb functional exercise, what do you think is the likelihood of postoperative complications? ) | RP1 | Breast cancer-related lymphedema | Keep |
|  | RP2 | Shoulder dysfunction | Keep |
|  | RP3 | [Subcutaneous exudate](C:/Program%20Files%20(x86)/Youdao/Dict/8.10.0.0/resultui/html/index.html#/javascript:;) | Keep |
|  | RP4 | Flap necrosis | Keep |
|  | RP5 | Muscle atrophy | Keep |
| Maintenance self-efficacy (MSE)  (I can maintain my upper limb functional exercise under the following conditions:) | MSE1 | Even if I feel tired | Keep |
|  | MSE2 | Even if I feel nervous and stressed | Keep |
|  | MSE3 | Even if I have to keep trying | Keep |
|  | MSE4 | Even if the function of my affected upper limb doesn't return soon | Keep |
|  | MSE5 | Even if it takes me a long time to form the habit of upper limb functional exercise | Keep |
|  | MSE6 | Even if my family and friends didn't urge me to exercise my upper limb | Keep |
| Action Planning (AP)  (I have planned:) | AP1 | the time for upper limb functional exercise | Keep |
|  | AP2 | the location of the upper limb functional exercise | Keep |
|  | AP3 | the type, degree, and duration of upper limb functional exercise | Keep |
|  | AP4 | how to do the upper limb functional exercise method | Keep |
|  | AP5 | I have planned a person to supervise the implementation of the upper limb functional exercise | NA |
| Coping Planning (CP) | CP1 | I have a way to deal with situations that might interfere with the upper limb functional exercise program | Keep |
|  | CP2 | If I'm in a situation where I might miss my upper limb exercise program, I have a way to deal with it. | Keep |
|  | CP3 | I have a way to deal with situations that might prevent an upper limb exercise program | Keep |
|  | CP4 | If there is a situation that may interrupt the upper limb functional exercise program, I can handle it. | Keep |
|  | CP5 | I have a way to deal with it if I suddenly feel sick during the upper limb functional exercise | Keep |
| Recovery self-efficacy (RSE)  (Under the following circumstances, I believe I can resume the upper limb functional exercise) | RSE1 | I've postponed a couple of times the upper limb functional exercise | Keep |
|  | RSE2 | I've been out of the upper limb functional exercise for weeks | Keep |
|  | RSE3 | I haven't seen any effects from the upper limb functional exercise yet | Keep |
|  | RSE4 | I have postoperative complications (e.g., breast cancer-related lymphedema) | Keep |
|  | RSE5 | I have so many things to do that I hardly have time for upper limb functional exercise | Keep |
| Attitude Behavior (AB) | AB1 | I think the time and effort spent on the upper limb functional exercise is worth it | Keep |
|  | AB2 | I believe that upper limb functional exercise can reduce the incidence of postoperative complications | Keep |
|  | AB3 | I think doing functional exercise can be harmful to the upper limb or the body | NA |
|  | AB4 | I think it only takes a few days to do upper limb functional exercise, not necessarily for a long time | Keep |
|  | AB5 | I think whether we need to carry out the upper limb functional exercise should be based on our situation | Keep |
| Subjective Norm (SN) | SN1 | I am willing to do upper limb functional exercise as instructed by the medical staff | Keep |
|  | SN2 | The behavior of the patient’s upper limb functional exercise affects me | Keep |
|  | SN3 | I can get the relevant knowledge of upper limb functional exercise from the medical staff | Keep |
|  | SN4 | My family and friends encourage and urge me to do upper limb functional exercise | Keep |
|  | SN5 | My family and friends think I should follow the medical staff's advice to exercise the upper limb | Keep |
| Perceived Behavioral control (PBC) | PBC1 | I have enough time and energy to do upper limb functional exercise every day | Keep |
|  | PBC2 | For me, it's easy to stick to the upper limb functional exercise for a long time | Keep |
|  | PBC3 | I will continue to do upper limb functional exercise without being urged to do so | Keep |
|  | PBC4 | I was able to communicate with the medical staff when I had problems with my upper limb | Keep |
|  | PBC5 | When I feel that the results of functional exercise are not good, I can actively seek suggestions for improvement | Keep |
| Behavioral Intention (BI)  (In the next three months) | BI1 | I ask my family to push me to do upper limb functional exercise | NA |
|  | BI2 | I will actively observe and evaluate the effect of upper limb functional exercise | Keep |
|  | BI3 | Give me enough time and energy to do upper limb functional exercise every day | Keep |
|  | BI4 | I will strictly follow the requirements of upper limb functional exercise | Keep |
|  | BI5 | I will take the initiative to learn the relevant knowledge of upper limb functional exercise | Keep |
| ULFE-in hospital (ULFE-IH) | ULFE-IH1 | I learned the specific methods and skills of upper limb functional exercise | Keep |
|  | ULFE-IH2 | I did a strict upper limb functional exercise | Keep |
|  | ULFE-IH3 | I have been ready to standardize the upper limb functional exercise after discharge | Keep |
| ULFE-maintenance (ULFE-M) | ULFE-M1 | I insist on eating, brushing my teeth, combing my hair, and doing other daily activities with my affected limb every day | NA |
|  | ULFE-M2 | I do upper limb functional exercise every day | Keep |
|  | ULFE-M3 | I often observe the recovery of upper limb function | Keep |
|  | ULFE-M4 | When I encounter difficulties during functional exercise, I will actively seek help from others | Keep |
|  | ULFE-M5 | I will protect the affected limb and try not to do anything harmful to the upper limb (such as avoid lifting heavy objects) | NA |

NA: These indicators were deleted due to low loading. Items 3, 4, and 5 of the attitude behavior dimension were negatively worded questions. ULFE: Upper Limb Functional Exercise.
