# Supplementary material for: Theoretical Explanation of Upper Limb Functional Exercise and Its Maintenance in Postoperative Patients With Breast Cancer
Source: Front Psychol. 2022 Jan 5;12:794777. doi: 10.3389/fpsyg.2021.794777 (PMC8766984; doi:10.3389/fpsyg.2021.794777)
Supplement: Supplementary file 2 [file Table_2.DOCX]

Supplementary Material

# Supplementary Tables

**Supplementary Table 2.** Validity of the questionnaire and measurement model indices

| Constructs | Item labels | Mean | SD | Loadings | CR | AVE |
| --- | --- | --- | --- | --- | --- | --- |
| Task self-efficacy (TSE) | TSE1 | 3.44 | 1.11 | 0.77 | 0.94 | 0.75 |
|  | TSE2 | 3.49 | 1.16 | 0.89 |  |  |
|  | TSE3 | 3.28 | 1.26 | 0.85 |  |  |
|  | TSE4 | 3.41 | 1.28 | 0.92 |  |  |
|  | TSE5 | 3.32 | 1.28 | 0.89 |  |  |
| Positive Outcome Expectations (POE) | POE1 | 3.51 | 1.10 | 0.84 | 0.93 | 0.78 |
|  | POE2 | 3.36 | 1.25 | 0.90 |  |  |
|  | POE3 | 3.72 | 1.04 | 0.91 |  |  |
|  | POE4 | 3.87 | 1.00 | 0.88 |  |  |
| Negative Outcome Expectations (NOE) | NOE1 | 3.07 | 1.22 | 0.94 | 0.94 | 0.89 |
|  | NOE2 | 3.36 | 1.22 | 0.95 |  |  |
| Risk Perception (RP) | RP1 | 3.53 | 1.18 | 0.89 | 0.97 | 0.85 |
|  | RP2 | 3.38 | 1.27 | 0.92 |  |  |
|  | RP3 | 3.30 | 1.32 | 0.93 |  |  |
|  | RP4 | 2.95 | 1.35 | 0.94 |  |  |
|  | RP5 | 3.00 | 1.39 | 0.94 |  |  |
| Maintenance self-efficacy (MSE) | MSE1 | 2.78 | 1.10 | 0.85 | 0.94 | 0.73 |
|  | MSE2 | 2.89 | 1.20 | 0.85 |  |  |
|  | MSE3 | 3.21 | 1.12 | 0.89 |  |  |
|  | MSE4 | 3.27 | 1.13 | 0.85 |  |  |
|  | MSE5 | 3.34 | 1.11 | 0.86 |  |  |
|  | MSE6 | 3.36 | 1.06 | 0.84 |  |  |
| Action Planning (AP) | AP1 | 2.95 | 1.16 | 0.92 | 0.95 | 0.82 |
|  | AP2 | 3.06 | 1.17 | 0.94 |  |  |
|  | AP3 | 3.25 | 1.12 | 0.91 |  |  |
|  | AP4 | 2.85 | 1.06 | 0.84 |  |  |
| Coping Planning (CP) | CP1 | 2.86 | 1.15 | 0.90 | 0.94 | 0.75 |
|  | CP2 | 2.58 | 1.21 | 0.92 |  |  |
|  | CP3 | 2.82 | 1.20 | 0.91 |  |  |
|  | CP4 | 2.63 | 1.15 | 0.90 |  |  |
|  | CP5 | 2.92 | 1.10 | 0.69 |  |  |
| Recovery self-efficacy (RSE) | RSE1 | 3.26 | 1.09 | 0.91 | 0.95 | 0.79 |
|  | RSE2 | 3.30 | 1.22 | 0.92 |  |  |
|  | RSE3 | 3.27 | 1.23 | 0.89 |  |  |
|  | RSE4 | 2.77 | 1.25 | 0.83 |  |  |
|  | RSE5 | 3.02 | 1.18 | 0.89 |  |  |
| Attitude Behavior (AB) | AB1 | 3.86 | 1.01 | 0.88 | 0.88 | 0.65 |
|  | AB2 | 3.79 | 1.03 | 0.87 |  |  |
|  | AB4 | 3.71 | 1.08 | 0.71 |  |  |
|  | AB5 | 3.44 | 1.13 | 0.75 |  |  |
| Subjective Norm (SN) | SN1 | 3.90 | 0.94 | 0.86 | 0.92 | 0.70 |
|  | SN2 | 3.54 | 1.25 | 0.82 |  |  |
|  | SN3 | 3.87 | 0.97 | 0.87 |  |  |
|  | SN4 | 3.62 | 1.17 | 0.79 |  |  |
|  | SN5 | 3.87 | 0.99 | 0.85 |  |  |
| Perceived Behavioral control (PBC) | PBC1 | 3.75 | 1.00 | 0.84 | 0.94 | 0.76 |
|  | PBC2 | 3.16 | 1.17 | 0.83 |  |  |
|  | PBC3 | 3.37 | 1.19 | 0.89 |  |  |
|  | PBC4 | 3.42 | 1.27 | 0.91 |  |  |
|  | PBC5 | 3.34 | 1.30 | 0.88 |  |  |
| Behavioral Intention (BI) | BI2 | 3.45 | 1.18 | 0.90 | 0.95 | 0.82 |
|  | BI3 | 3.68 | 1.09 | 0.89 |  |  |
|  | BI4 | 3.64 | 1.16 | 0.91 |  |  |
|  | BI5 | 3.41 | 1.34 | 0.91 |  |  |
| ULFE-in hospital (ULFE-IH) | ULFE-IH1 | 3.64 | 1.06 | 0.93 | 0.94 | 0.83 |
|  | ULFE-IH2 | 3.79 | 1.02 | 0.94 |  |  |
|  | ULFE-IH3 | 3.34 | 1.31 | 0.87 |  |  |
| ULFE-maintenance (ULFE-M) | ULFE-M2 | 3.36 | 1.37 | 0.84 | 0.83 | 0.63 |
|  | ULFE-M3 | 3.25 | 1.09 | 0.76 |  |  |
|  | ULFE-M4 | 2.93 | 1.08 | 0.77 |  |  |

ULFE: Upper Limb Functional Exercise; CR: composite reliability; AVE: average variance extracted.
