# Supplementary material for: Theoretical Explanation of Upper Limb Functional Exercise and Its Maintenance in Postoperative Patients With Breast Cancer
Source: Front Psychol. 2022 Jan 5;12:794777. doi: 10.3389/fpsyg.2021.794777 (PMC8766984; doi:10.3389/fpsyg.2021.794777)
Supplement: Supplementary file 3 [file Table_3.DOCX]

Supplementary Material

# Supplementary Tables

**Supplementary Table 3.** Discriminant validity: Fornell–Larcker criterion

| Fornell-Larcker Criterion | AB | AP | BI | CP | ULFE-IH | ULFE-M | MSE | NOE | PBC | POE | RP | RSE | SN | TSE |
| --- | --- | --- | --- | --- | --- | --- | --- | --- | --- | --- | --- | --- | --- | --- |
| Attitude Behavior (AB) | **0.81** |  |  |  |  |  |  |  |  |  |  |  |  |  |
| Action Planning (AP) | -0.07 | **0.90** |  |  |  |  |  |  |  |  |  |  |  |  |
| Behavioral Intention (BI) | 0.67 | 0.14 | **0.90** |  |  |  |  |  |  |  |  |  |  |  |
| Coping Planning (CP) | 0.01 | 0.75 | 0.17 | **0.87** |  |  |  |  |  |  |  |  |  |  |
| ULFE-in hospital (ULFE-IH) | 0.70 | 0.07 | 0.81 | 0.12 | **0.91** |  |  |  |  |  |  |  |  |  |
| ULFE-maintenance (ULFE-M) | -0.14 | 0.77 | 0.07 | 0.72 | 0.02 | **0.79** |  |  |  |  |  |  |  |  |
| Maintenance self-efficacy (MSE) | -0.05 | 0.80 | 0.14 | 0.75 | 0.05 | 0.77 | **0.86** |  |  |  |  |  |  |  |
| Negative Outcome Expectations (NOE) | 0.45 | -0.10 | 0.39 | -0.12 | 0.42 | -0.20 | -0.09 | **0.94** |  |  |  |  |  |  |
| Perceived Behavioral Control (PBC) | 0.74 | 0.07 | 0.86 | 0.10 | 0.82 | 0.03 | 0.07 | 0.41 | **0.87** |  |  |  |  |  |
| Positive Outcome Expectations (POE) | 0.73 | -0.05 | 0.62 | 0.01 | 0.63 | -0.10 | -0.04 | 0.53 | 0.66 | **0.88** |  |  |  |  |
| Risk Perception (RP) | 0.58 | -0.07 | 0.52 | -0.04 | 0.53 | -0.14 | -0.07 | 0.54 | 0.53 | 0.58 | **0.92** |  |  |  |
| Recovery self-efficacy (RSE) | -0.07 | 0.62 | 0.05 | 0.57 | 0.02 | 0.61 | 0.67 | -0.08 | 0.002 | -0.10 | -0.09 | **0.89** |  |  |
| Subjective Norm (SN) | 0.76 | 0.05 | 0.80 | 0.08 | 0.78 | -0.04 | 0.05 | 0.52 | 0.79 | 0.70 | 0.58 | -0.03 | **0.84** |  |
| Task self-efficacy (TSE) | 0.69 | -0.02 | 0.62 | 0.03 | 0.71 | -0.07 | -0.05 | 0.51 | 0.68 | 0.70 | 0.58 | -0.10 | 0.66 | **0.87** |

ULFE: Upper Limb Functional Exercise.
