# Supplementary material for: Theoretical Explanation of Upper Limb Functional Exercise and Its Maintenance in Postoperative Patients With Breast Cancer
Source: Front Psychol. 2022 Jan 5;12:794777. doi: 10.3389/fpsyg.2021.794777 (PMC8766984; doi:10.3389/fpsyg.2021.794777)
Supplement: Supplementary file 4 [file Table_4.DOCX]

Supplementary Material

# Supplementary Tables

**Supplementary Table 4.** Results of R^2^, Q^2^

| Constructs | R^2^ | Q^2^ |
| --- | --- | --- |
| Action Planning (AP) | 0.64 | 0.49 |
| Behavioral Intention (BI) | 0.77 | 0.59 |
| Coping Planning (CP) | 0.57 | 0.40 |
| ULFE-in hospital (ULFE-IH) | 0.72 | 0.57 |
| ULFE-maintenance (ULFE-M) | 0.68 | 0.38 |
| Recovery self-efficacy (RSE) | 0.45 | 0.33 |

ULFE: Upper Limb Functional Exercise.
